# 82 Early Skin Excision Decreased the Risk of Skin Infection, Sepsis and Mortality Among Burn Patients

**DOI:** 10.1093/jbcr/irac012.085

**Published:** 2022-03-23

**Authors:** Giovanna De La Tejera, Juquan Song, Kassandra K Corona, Sunny Gotewal, Kendall Wermine, Tsola A Efejuku, Phillip H Keys, Alejandro A Joglar, Elvia L Villarreal, Lyndon G Huang, Shivan N Chokshi, Jasmine M Chaij, Shelby P Bagby, Maria Haseem, George Golovko, Amina El Ayadi, Steven E Wolf

**Affiliations:** University of Texas Medical Branch, Galveston, Texas; University of Texas Medical Branch, Galveston, Texas; University of Texas Medical Branch, Galveston, Texas; University of Texas Medical Branch, Galveston, Texas; University of Texas Medical Branch, Galveston, Texas; University of Texas Medical Branch, Galveston, Texas; University of Texas Medical Branch, Galveston, Texas; University of Texas Medical Branch, Galveston, Texas; University of Texas Medical Branch, Galveston, Texas; University of Texas Medical Branch, Galveston, Texas; University of Texas Medical Branch at Galveston, Galveston, Texas; University of Texas Medical Branch, Galveston, Texas; University of Texas Medical Branch, Galveston, Texas; University of Texas Medical Branch, Galveston, Texas; University of Texas Medical Branch at Galveston, Galveston, Texas; University of Texas Medical Branch at Galveston, Galveston, Texas; University of Texas Medical Branch at Galveston, Galveston, Texas

## Abstract

**Introduction:**

In lieu of outdated and limited patient studies on excision for severe burns, a more comprehensive analysis is indicated to determine the effects of early skin excision following burn. This study aims to address the outcomes of early excision.

**Methods:**

Data collection and analysis was performed using TriNetX, a national research database. The study population included patients ranging from 0 to 90 years old who underwent excision for burns. Groups were stratified by the number of days after injury in which they received a skin excision treatment. Five outcomes were analyzed: death, cardiac stress, wound infection, blood transfusion, and sepsis. Risk and incidence of various health outcomes were compared between groups after matching for age, gender and race, using a z-test with p< 0.05 considered significant.

**Results:**

We identified 2,522 patients who underwent excision between 0-3 days, 825 between 4-7 days, and 419 between 8-14 days following burn. We found a significant decrease in risk of skin infection and sepsis for skin excision 0-3 days after burn compared to 4-7 days (p< 0.05). Additionally, the frequency of blood transfusion significantly increased for those with excision 0-3 days after burn when compared to 4-7 days (p< 0.05). There was a significant increase in the risk of mortality for patients who received skin excision 8-14 days after injury as compared to both 0-3 days (p< 0.05) and 4-7 days (p< 0.05). However, we found no statistical difference in cardiac stress, skin infection, blood transfusion or sepsis between 0-3 and 8-14 days nor 4-7 and 8-14 days.

**Conclusions:**

Skin excision 0-3 days after burn injury results in a significantly lowered risk of skin infection and sepsis as compared to skin excision 4-7 days and 8-14 days after burn. Skin excision within the first 7 days after burn decreased the risk of mortality as compared to excision 8-14 days after burn. The risk of blood transfusion increased with early excision, which may be explained by the severity of the injury.

